# Whole genome sequencing of Asia II 1 species of whitefly reveals that genes involved in virus transmission and insecticide resistance have genetic variances between Asia II 1 and MEAM1 species

**DOI:** 10.1186/s12864-019-5877-9

**Published:** 2019-06-18

**Authors:** Sonia Hussain, Muhammad Farooq, Hassan Jamil Malik, Imran Amin, Brian E. Scheffler, Jodi A. Scheffler, Shu-Sheng Liu, Shahid Mansoor

**Affiliations:** 1 National Institute for Biotechnology and Genetic Engineering, Faisalabad, Pakistan; 2 Department of Biotechnology, Pakistan Institute of Engineering & Applied Sciences (PIEAS), Nilore, Islamabad, Pakistan; 3 USDA-ARS, Genomics and Bioinformatics Research Unit, 141 Experiment Station Rd., Stoneville, MS 38776 USA; 4 USDA-ARS, Crop Genetics Research Unit, 141 Experiment Station Rd, Stoneville, MS 38776 USA; 5 Institute of Insect Sciences, College of Agriculture and Biotechnology, Zhejiang University, Hangzhou, 310058 China

**Keywords:** Whitefly, Asia II 1, MEAM1, Sequencing, Virus, Insecticide

## Abstract

**Background:**

Whiteflies (*Bemisia tabaci*) are phloem sap-sucking pests that because of their broad host range and ability to transmit viruses damage crop plants worldwide. *B. tabaci* are now known to be a complex of cryptic species that differ from each other in many characteristics such as mode of interaction with viruses, invasiveness, and resistance to insecticides. Asia II 1 is an indigenous species found on the Indian sub-continent and south-east Asia while the species named as Middle East Asia Minor 1 (MEAM1), likely originated from the Middle-East and has spread worldwide in recent decades. The purpose of this study is to find genomic differences between these two species.

**Results:**

Sequencing of the nuclear genome of Asia II 1 with Illumina HiSeq and MiSeq generated 198.90 million reads that covers 88% of the reference genome. The sequence comparison with MEAM1 identified 2,327,972 SNPs and 202,479 INDELs. In Total, 1294 genes were detected with high impact variants. The functional analysis revealed that some of the genes are involved in virus transmission including 4 genes in *Tomato yellow leaf curl virus* (TYLCV) transmission, 96 in *Tomato crinivirus* (ToCV) transmission, and 14 genes in insecticide resistance.

**Conclusions:**

These genetic differences between Asia II 1 and MEAM1 may underlie the major biological differences between the two species such as virus transmission, insecticide resistance, and range of host plants. The present study provides new genomic data and information resources for Asia II 1 that will not only contribute to the species delimitation of whitefly, but also help in conceiving future research studies to develop more targeted management strategies against whitefly.

**Electronic supplementary material:**

The online version of this article (10.1186/s12864-019-5877-9) contains supplementary material, which is available to authorized users.

## Introduction

*Bemisia tabaci* (Hemiptera: *Aleyrodidae*), commonly known as ‘whiteflies’ are phloem sap sucking pests some of which have become a major constraint to important food, fiber and ornamental crops worldwide. The whiteflies can infest as many as 1000 plant species [1] and they damage host plants by infestation, but more importantly by transmitting plant viruses. These whiteflies can potentially vector over 300 plant viruses, mostly viruses in the genus *Begomovirus* [2]. Major crops affected by *B. tabaci*-transmitted viruses on a global scale include cotton, cassava, tomato, sweet potato, cucurbits and other crop plant species.

Whiteflies (*B. tabaci*) are now known to be as a cryptic species complex, based on recent molecular phylogenetic analyses and evidence of reproductive incompatibility [3, 4]. These putative whitefly species differ in many biological aspects such as host range [1], resistance to insecticides [5, 6], specificity and capacity of virus transmission [7, 8] and composition of harbored symbionts [9]. Although the use of ≥3.5% mtCOI divergence as the criterion for species delimitation has been occasionally shown to be inadequate [10], it has been widely used to differentiate species. Based on sequence divergence of mtCOI (≥ 3.5% divergence), *B. tabaci* has been deduced to include more than 39 cryptic species that are morphologically indistinguishable but genetically distinct [11–13].

The long-term association between begomoviruses and whitefly has brought some co-evolved adaptations [14] that allow them to live in equilibrium. Begomoviruses are single-stranded (ss) DNA viruses that are transmitted mostly in a persistent circulative manner. Once ingested through the stylet, these plant viruses move across the mid gut membrane and then via hemolymph translocate to salivary glands and from there these are egested while feeding [15]. In circulation of viruses, mid gut and salivary glands are the main barriers to overcome [16, 17]. Some mid gut proteins and proteins produced by endosymbionts in hemolymph are associated with circulation of viruses in whitefly. These interacting proteins are the main points which lead to the differentiation of cryptic species on the basis of specificity and capacity of virus transmission. The heat shock protein HSP70 is co-localized with *Tomato yellow leaf curl virus* (TYLCV) coat protein within midgut epithelial cells and inhibits virus transmission [18]. Knottin-1 restricts the virus (TYLCV) amount in whitefly and thus shields the whitefly against its deleterious effects [19]. While cyclophilin B enhances the translocation of virus from mid gut to hemolymph [20]. Another protein peptidoglycan recognition protein (*Bt*PRPG) is involved in whitefly immunity and has a potential binding site for TYLCV. Its co-localization with TYLCV is also reported within the midgut [21]. Endosymbionts which have been living in whitefly for millions of years [22] are also involved in virus transmission. Different cryptic species harbor different endosymbionts. Endosymbionts reside in bacteriocytes and some of them (e.g. *Hamiltonella*) produce GroEL homologue in the hemolymph which helps in virus circulation in whitefly.

Middle East-Asia Minor 1 (MEAM1, formerly known as “biotype B”) and Mediterranean (MED, formerly “Q biotype”) are globally important cryptic species of whitefly [23, 24] because of their invasiveness and broad host range. The two species originated in the Middle East regions, but are now reported from many regions of the world, and its presence has also been well reported in the southern Sindh region of Pakistan [25, 26]. Asia 1 and Asia II 1 are two species of whitefly indigenous to Pakistan, with Asia II 1 being the most prevalent whitefly in the central region of the country [26]. The different species of whitefly recorded from Pakistan have been shown to differ in many aspects including virus transmission, insecticide resistance, and host range. For example, MEAM1 is more efficient than Asia II 1 in transmitting *Tomato yellow leaf curl virus* (TYLCV) [27]. In a study in Vietnam where Asia II 1 is indigenous, Asia II 1 is reported to be more efficient in transmitting *Tomato leaf curl Hainan virus* (ToLCHnV) than that of TYLCV, while MEAM1 is more efficient in transmitting TYLCV than ToLCHnV [28]. Asia II 1 has been reported to be the most abundant species of whitefly in areas of high incidence of cotton leaf curl disease (CLCuD) in Pakistan and the western region of India. Two recent studies in China [17, 29] directly compared the transmission efficiency of begomoviruses by MEAM1, Asia II 1 and two more species, and showed that among these species Asia II 1 is the most efficient in transmitting both *Cotton leaf curl Multan virus* (CLCuMuV) and *Tobacco curly shoot virus* (TbCSV). Apart from differences in transmission efficiency of viruses, these species of whiteflies also differ in insecticide resistance [30] and host plant preference [31]. However, the physiological and molecular mechanisms underlying the differences between species of whitefly are yet poorly known.

Over the past several years, next generation sequencing (NGS) technology has emerged as an innovative approach to high-throughput sequencing [32], and the rapid development of this modern technology provides us an unprecedented opportunity to understand and explore numerous genetic findings, which can help to improve our research on the physiology and molecular biology of the whiteflies. These results can also provide new knowledge and concepts for the development of novel strategies and technology to manage whitefly pests and the viral disease agents they vector. In this study, our aim is to unravel some genetic information from Asia II 1 and MEAM1, the two major whitefly pests in Pakistan. First, with access to the data of MEAM1 [4], we performed high throughput sequencing of Asia II 1 and aligned with that of MEAM1, to identify major genomic differences between the two species. We detected some high impact variants in genes (which were previously reported as differentially expressed genes) that have been predicted to be associated with virus transmission and insecticide resistance.

## Results

### Mapping summary of nuclear genome

**Fig. 1:**
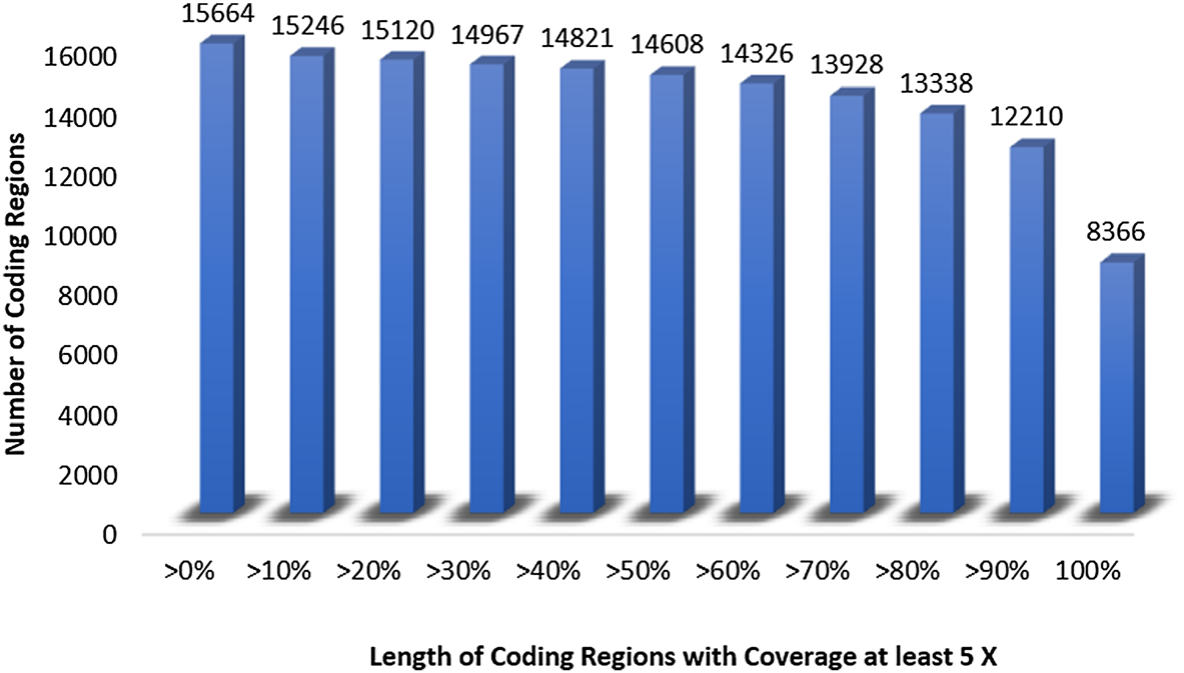
Total coding regions are 15,664. All the coding regions with less than 10% each of their length are covered with at least 5X coverage, 53% of coding regions (8366) with full length are covered with at least 5X coverage

Genome sequencing of Asia II 1 with Illumina HiSeq and MiSeq generated a total of 31.15 Gb of data comprising 198.90 million reads with read size 100 and 300 bp (the summary of raw data generated from each of seven libraries is given in Additional file 1). Approximately 91% of the reads passed the quality control criteria and 82 to 86% of these reads were mapped correctly to the reference genome. The available sequence from the reference genome is 615 Mb [4] of the assessed total genome size of ~ 680–690 Mb as estimated in a previous study [33] using both flow cytometry and Kmer analysis. These reads covered 88% of the reference genome. The mean read length was 159 bp. The summary of the sequencing and mapping is shown in Table 1. The average depth of coverage of genome after filtration was 34X. Total length of the coding region of the reference genome is 44.43 Mb, 51% of which was covered with more than 5X depth of coverage, and 53% of the number of coding regions with 100% of length have at least 5X depth of coverage. The mean coverage of the coding region is 32X. Figure 1 displays the different number of coding regions with different lengths having at least 5X depth of coverage. Approximately 8366 coding regions have at least 5X coverage with full length genes.

**Table 1:** Mapping Summary

Total NGS Library	7
Total Insert Size	550
Sequencer	IlluminaHiSeq2500 &MiSeq
Total Raw Data Generate	(HiSeq: 14GB)
	(MiSeq: 16 Gb)
	Total: 31.15 Gb
Average Coverage	47.34 X
Average Coverage After Filtration	34.52 X
Total No of Reads Generate	HiSeq: 142605246
	MiSeq: 56300942
	Total: 198906188
Total No of Reads Quality Passed	181,434,767
Total No of Reads Mapped	156,293,812 (86%)
Total No of Reads Mapped Properly	149,439,368 (82%)
Reference Genome Covered	88%
Mean Read Length	159 bp

### Variant statistics

After variant calling and two times filtration with Genome Analysis Tool Kit (GATK), total number of 2,530,451 high quality variants were discovered. Variant annotations and effect prediction through SnpEff resulted in 3,504,011 effects. Effects are greater in number than number of variants as one variant could have more than one effect. For example, one variant could be non-synonymous for one gene while being downstream to another. A variant statistics summary is given in Table 2 (raw variant calling data and the data after each filtration is provided in Additional file 2). Approximately 2,327,972 SNPs and 202,479 INDELs were detected. In eight amplified regions ranging in size from 500 to 600 bp, there are 96 SNPs which were all validated through Sanger sequencing. The primers pairs list and validated SNPs positions are given in Additional file 4. The initial average variant rate was 1/20 bp, but that was decreased to 1/235 bp after filtration (when depth of read coverage at a variant point was increased to 30X in variant calling criterion). Variant rate also varied in different regions, the maximum variant rate recorded was 1/27 bp and minimum variant rate was 1/32,808 bp. Transition to transversion ratio is 1.71 and heterozygous to homozygous variant ratio is 0.05. In this study, insertions and deletions ranging from 1 to 100 bp were considered as INDELs. The maximum number of INDELs were 1 bp in length while lowest number of INDELs were of 14, 15, 20, 21, 23, 28, 33, 69, 89 or 100 bp in length. The distribution and types of variant effects in the whole genome are given in Table 3. According to functional effects of variants, these were distributed into three classes; silent (69.94%), missense (29.77%) and nonsense (0.29%).

**Table 2:** Variant Statistics

Number of variants	2,530,451
Number of effects	3,504,011
Variant rate	1 /235 bases
SNP	2,327,972
INS	103,960
DEL	98,519
Missense / Silent	0.4257
Ts/Tv ratio	1.7147
Heterozygous	122,045
Homozygous	2,349,906
Heterozygous/Homozygous	0.05193612

**Table 3:** Classification of effects and their number in the whole genome

Type of Effects		No of Effects	
		Count	Percent
High Effect	Total	1821	0.052
Splice acceptor variant		96	0.003
Splice donor variant		135	0.004
Start loss		56	0.002
Stop gain		371	0.003
Stop lost		96	0.003
Frame shift		1102	0.031
Moderate Effect	Total	35,583	2.724
Conservative inframe deletion		49	0.001
Conservative inframe insertion		83	0.002
Disruptive inframe deletion		98	0.003
Disruptive inframe insertion		74	0.002
Missense variant		35,285	1.004
Low Effect	Total	95,439	1.015
5′ UTR premature start gain		3020	0.086
Splice region variant		10,980	0.312
Stop retained		106	0.003
Synonymous variant		83,150	2.366
Initiator codon/ non syn start		15	0
Modifier Effects	Total	3,371,168	96.209
3′ UTR		174,811	4.974
5′ UTR		23,577	0.671
Downstream		485,837	13.823
Upstream		421,908	12.041
Non-coding transcript		470	0.013
Intron variant		1,479,087	42.082
Intergenic regions		794,375	22.67

**Fig. 2:**
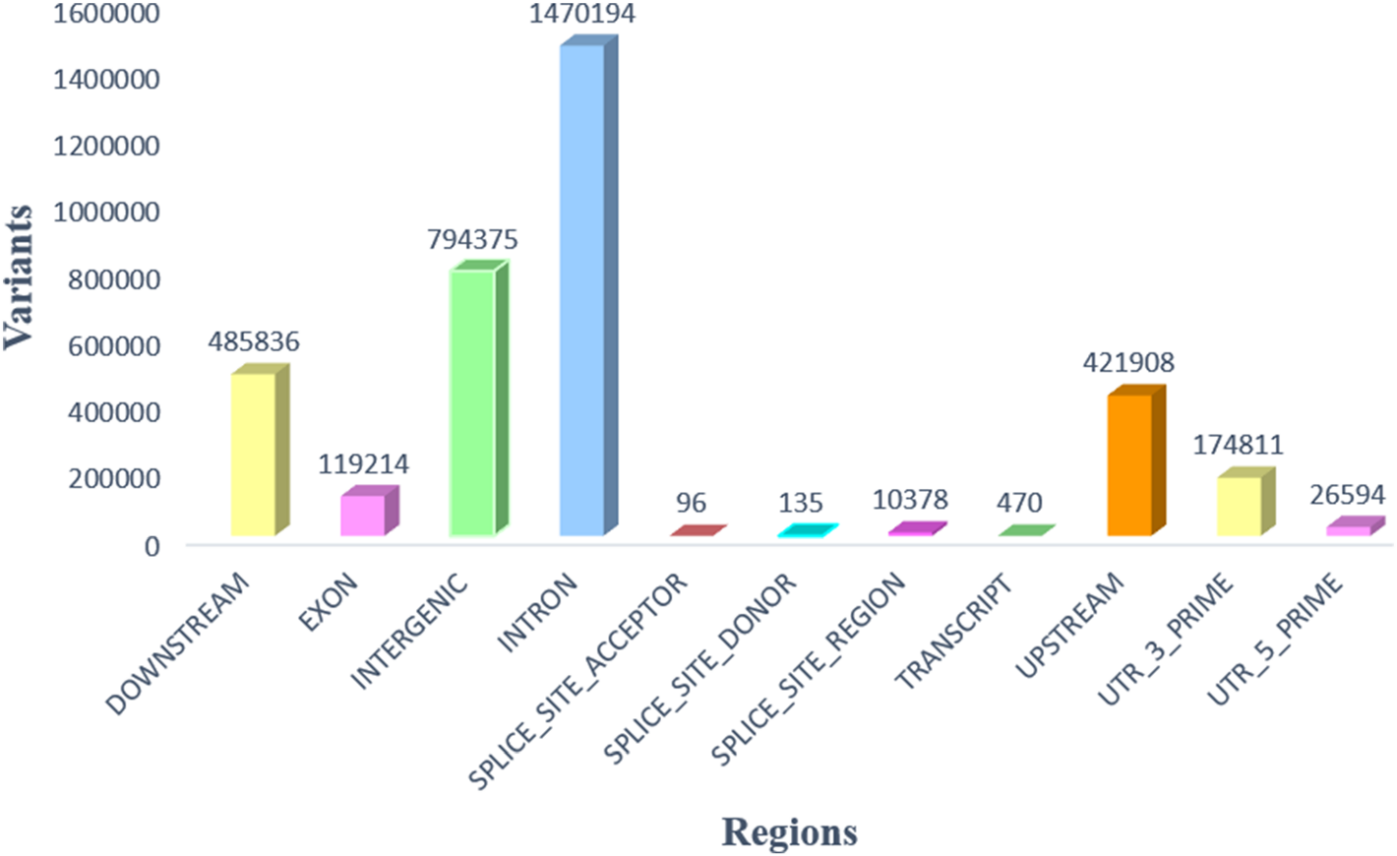
Distribution of variants in different genic regions

Among the total estimated genes in whitefly MEAM1 (15,664), 1294 genes were found to have high impact variants in this data. These genes were selected for further analysis of ontology. The distribution and number of variants and their effects in different genic regions are given in Fig. 2. The number of genes in each class of high impact variants are also provided in Table 4.

**Table 4:** Number of variant genes in each sub-class of high effects. One gene may have more than one effect and same gene may count in more than one category of high effects

Type of High Effects	No of Genes
Splice acceptor variant	92
Splice donor variant	129
Start Loss	55
Stop gain	346
Stop lost	91
Frame shift	765
Total	1294

### Gene ontology

**Fig. 3:**
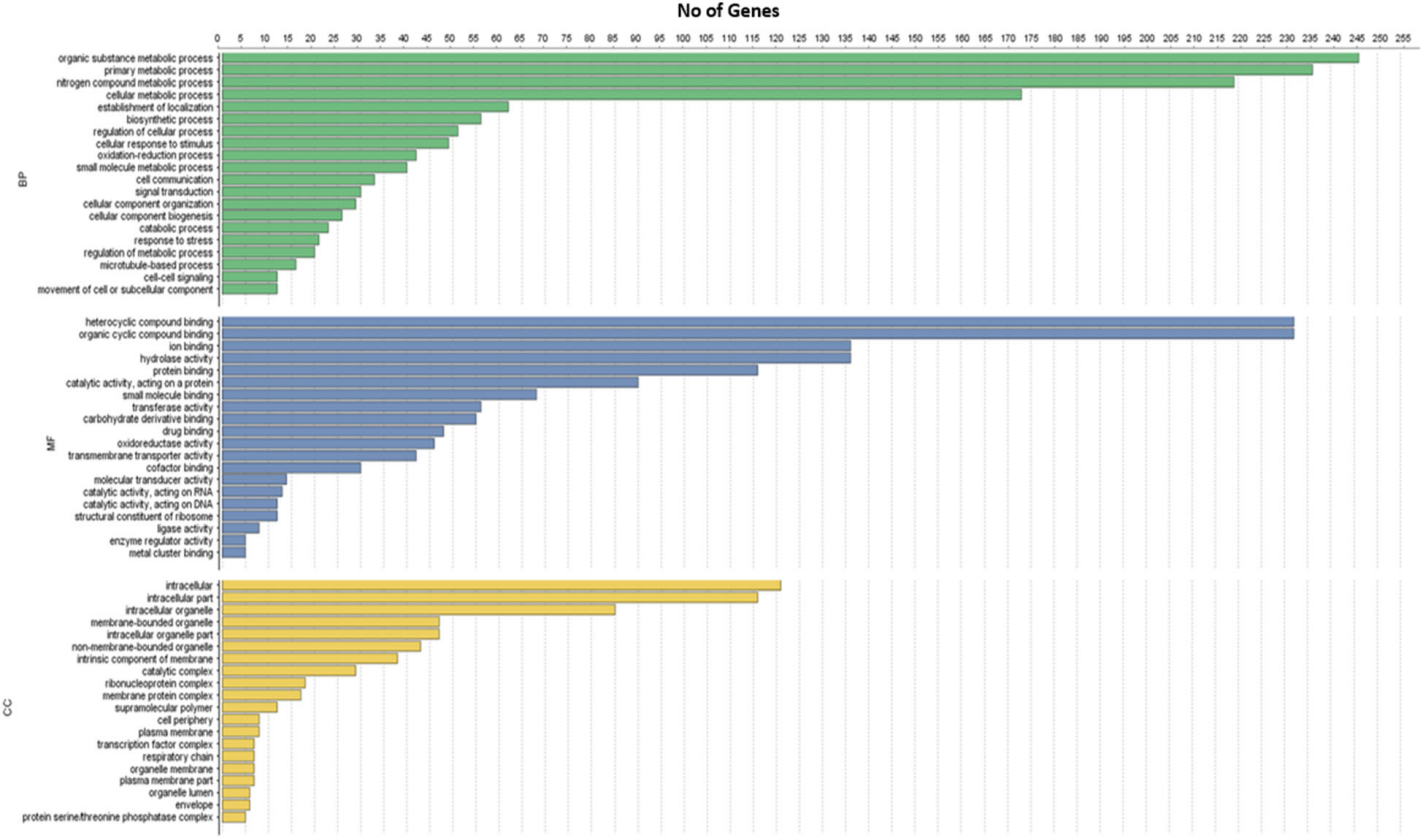
Histogram representation of GO classification of genes with high impact variants. These genes are classified into CC: cellular component, BP: biological process and MF: molecular function. In the supplementary data, genes are listed, that belong to each of sub class of these three categories

Coding regions that have high impact variants (1294 genes) were selected for gene ontology analysis. The Blast2GO results are shown in Fig. 3. The functions of these were classified into three groups: biological process (BP), molecular function (MF), and cellular components (CC). The greatest number of genes were associated with the BP category. IDs of genes associated with each sub category of these three functional classes are given in Additional file 3. Additional file 7 shows the associated pathways for the genes (with high impact variants), which were predicted by Blast2GO.

### Genes involved in virus transmission and insecticide resistance

Fourteen genes of MEAM1 that were reported for potential involvement in insecticide resistance [4] and 96 genes which were reported to be associated with virus transmission [6] were expected to be high impact variants between Asia II 1 and MEAM1. In the present study, there were 15 high impact variants found in 14 genes which could potentially be involved in insecticide resistance. High impact variants include frame shift, start loss, stop gain, splice acceptor and splice donor. These lead to truncated or modified proteins with partial or complete loss of function. There is also a chance that because of these mutations some of the proteins may gain more efficiency rather than to be dis-functional. These 14 genes belong to 4 gene families: acetylcholinesterase like protein, cathepsin (B, F, cathepsin L like), Cytochrome P450, and phosphatidylethanolamine-binding protein 1. A list of these insecticide resistance gene IDs is shown in Table 5 and those for virus transmission in Table 6 (TYLCV) and 7 (ToCV). All the genes described in Table 5 are reported for the potential involvement in insecticide resistance by Chen et al.*,* [4], and those for virus transmission in Table 6 and Table 7 are reported by Hasegawa et al.*,* [34] and Kaur et al.*,* [6] respectively.

**Table 5:** Genes potentially involved in insecticide resistance with variants between Asia II 1 and MEAM1

Gene ID	Annotation	Type of Variant	Variant Position
Bta08717	Acetylcholinesterase-like protein	Frame Shift	Scaffold325:2419087
Bta12286	Cathepsin B	start lost	Scaffold562:2252138
Bta06690	Cathepsin F	stop gain	Scaffold2605:1316025
Bta07152	Cathepsin L-like protease	Frame Shift	Scaffold2737:56518
Bta02560	Cathepsin L-like protease	Frame Shift	Scaffold132:3684567
Bta04696	Cytochrome P450	Splice acceptor	Scaffold1685:811440
Bta06044	Cytochrome P450	Stop lost	Scaffold231:1494714
Bta01556	Phosphatidylethanolamine-binding protein	Frame Shift	Scaffold1224:613594
Bta01355	Phosphatidylethanolamine-binding protein 1	start lost, splice acceptor variant	Scaffold1195:116803, Scaffold1195:118926
Bta15207	Phosphatidylethanolamine-binding protein 1	Start lost	Scaffold923:587527
Bta07891	Phosphatidylethanolamine-binding protein 1	splice donor	Scaffold300:6735496
Bta12136	Phosphatidylethanolamine-binding protein 1	Frame Shift	Scaffold545:18333
Bta13188	Phosphatidylethanolamine-binding protein 1	Splice acceptor	Scaffold637:1563358
Bta02907	Phosphatidylethanolamine-binding protein, putative	Frame Shift	Scaffold14:2449776

**Table 6:** List of gene IDs which are potentially involved in TYLCV virus transmission and have genetic variants between Asia II 1 and MEAM1

Gene ID	Annotation	Type of Variant	Variant Position
Bta10341	Aldo-keto reductase	Frame Shift	Scaffold 403:3624744
Bta04072	Elicitin-like protein 6	Frame Shift	Scaffold161:5952976
Bta02276	Ubiquitin carboxyl-terminal hydrolase	Frame Shift	Scaffold130:858376
Bta14634	Unknown protein	Frame Shift, Splice Donor Variant	scaffold811: 176696, Scaffold811:176710

**Table 7:** List of gene IDs which are potentially involved in ToCV virus transmission and have genetic variants between Asia II 1 and MEAM 1

Gene ID	Gene Name	Type of Variants	Scaffold:Snp Position
Bta08892	70 kDa heat shock protein	Frame shift	Scaffold3328:264318
Bta01665	AAA-ATPase-like domain-containing protein	frame shift	Scaffold1224:5022892
Bta12603	AAA-ATPase-like domain-containing protein	stop gain	Scaffold597:2078628
Bta05346	Afadin, putative	stop lost	Scaffold199:1272506
Bta11978	Alpha-glucosidase	Frame shift	Scaffold521:859550
Bta01804	Ankyrin repeat and LEM domain-containing protein	Frame shift	Scaffold123:4405622
Bta01772	Cathepsin B	Frame shift	Scaffold123:2832350
Bta07402	Cathepsin B	Frame shift	Scaffold2816:1342943
Bta02120	Cathepsin L-like protease	Frame shift	Scaffold1261:554552
Bta02560	Cathepsin L-like protease	Frame shift	Scaffold132:3684567
Bta07152	Cathepsin L-like protease	Frame shift	Scaffold2737:56518
Bta06739	Cation transport regulator-like protein 1	Frame shift	Scaffold2605:2569958
Bta08022	CG13675, isoform D	Frame shift	Scaffold3040:3058531
Bta04412	CG14375	Frame shift	Scaffold165:195426
Bta03710	CG17612, isoform A	splice acceptor variant	Scaffold155:194033
Bta11746	CG7120, isoform F	splice donor, frame shift	Scaffold52:4009995
Bta10928	Chromodomain Y-like protein 2	Frame shift	Scaffold477:1214758
Bta12891	Citron Rho-interacting kinase	Frame shift	Scaffold613:2332964
Bta02184	Cystatin	frame shift	Scaffold128:1309680
Bta07162	DDB1-and CUL4-associated factor	start loss,stop gain,	Scaffold2737:410077, Scaffold2737:419749
Bta07434	DNA-directed RNA polymerase, omega subunit family protein	stop gain, frame shift	Scaffold2890:209245
Bta14689	Dolichyl-diphosphooligosaccharide--protein glycosyltransferase subunit STT3B	splice acceptor variant	Scaffold811:2521172
Bta15680	E3 ubiquitin-protein ligase TTC3	Frame shift	Scaffold988:3252798
Bta03681	Eukaryotic translation initiation factor 3 subunit A	Frame shift	Scaffold1512:1481746
Bta14560	Galectin	Frame shift	Scaffold809:3964391
Bta10009	General transcription factor 3C polypeptide 2	Frame shift	Scaffold382:2610001
Bta04387	GH16255p	splice acceptor variant	Scaffold1647:2597569
Bta00770	GK11989	stop lost, frame shift	Scaffold1103:753116
Bta01833	Klarsicht, isoform E	stop gain, frame shift	Scaffold123:5600056
Bta09051	Laminin subunit beta-1	stop gain	Scaffold338:1218247
Bta01704	Loquacious	Frame shift	Scaffold123:255863
Bta03800	Lysosomal-trafficking regulator	Frame shift	Scaffold155:4253425
Bta05467	Major royal jelly-related protein	stop lost	Scaffold199:6871677
Bta05773	NADH dehydrogenase [ubiquinone] 1 alpha subcomplex subunit 12	stop gain	Scaffold2229:151223
Bta15454	Neuroendocrine convertase 1	Frame shift	Scaffold959:3096344
Bta10191	Nidogen-2	stop gain	Scaffold3978:2723
Bta13257	Protein patched	Frame shift	Scaffold641:259689
Bta15368	Protein phosphatase 1 L	Frame shift	Scaffold959:15812
Bta13589	Protein unc-45-like protein A	Frame shift	Scaffold651:2187902
Bta02051	Regucalcin	start loss	Scaffold1240:111531
Bta10926	Replication factor-a protein 1	Frame shift	Scaffold477:1149858
Bta12190	Sortilin-related receptor	Frame shift	Scaffold545:2426274
Bta02847	Sulfotransferase	stop gain	Scaffold14:67127
Bta08229	Symplekin	splice acceptor variant	Scaffold317:609334
Bta07946	Terribly reduced optic lobes, isoform AN	splice acceptor	Scaffold3040:556936
Bta05242	Transcriptional protein SWT1	Frame shift	Scaffold1898:321532
Bta09856	Trehalase	stop gain	Scaffold374:3016858
Bta03298	Trypsin-like serine protease	stop lost	Scaffold147:7182519
Bta09090	Tudor domain protein	stop lost	Scaffold338:1990664
Bta08596	Tudor domain-containing protein 1	Frame shift	Scaffold322:4722919
Bta03892	Ubiquitin carboxyl-terminal hydrolase	start lost	Scaffold1580:568946
Bta01518	Unknown protein	stop gain	Scaffold1214:734963
Bta01571	Unknown protein	Frame shift	Scaffold1224:1139169
Bta01615	Unknown protein	Frame shift	Scaffold1224:3224397
Bta02665	Unknown protein	Frame shift	Scaffold1339:520464
Bta02767	Unknown protein	Frame shift	Scaffold137:1379435
Bta02836	Unknown protein	Frame shift	Scaffold139:1098948
Bta02920	Unknown protein	Frame shift	Scaffold14:3202002
Bta03301	Unknown protein	stop gain, frame shift	Scaffold147:7328434
Bta03426	Unknown protein	stop gain	Scaffold1496:690294
Bta03435	Unknown protein	Frame shift	Scaffold1496:1047497
Bta04551	Unknown protein	Frame shift	Scaffold165:5163918
Bta04829	Unknown protein	Frame shift	Scaffold17:652047
Bta04921	Unknown protein	Frame shift	Scaffold17:652047
Bta05143	Unknown protein	stop gain	Scaffold18461:1072084
Bta05268	Unknown protein	stop gain, frame shift	Scaffold1971:32055
Bta05546	Unknown protein	Frame shift	Scaffold2013:237841
Bta05683	Unknown protein	Frame shift	Scaffold2124:427571
Bta05758	Unknown protein	Frame shift	Scaffold2225:1204179
Bta05761	Unknown protein	stop gain	Scaffold2225:1258041
Bta05893	Unknown protein	stop gain	Scaffold226:1397519
Bta06123	Unknown protein	splice donor	Scaffold231:3876649
Bta07727	Unknown protein	Frame shift	Scaffold300:708005
Bta07839	Unknown protein	stop gain	Scaffold300:4527825
Bta08000	Unknown protein	stop gain, frame shift	Scaffold3040:2567504
Bta08242	Unknown protein	Frame shift	Scaffold317:1074159
Bta08287	Unknown protein	stop gain	Scaffold320:265593
Bta08375	Unknown protein	stop gain	Scaffold320:3813827
Bta08462	Unknown protein	Frame shift	Scaffold322:385722
Bta08745	Unknown protein	Frame shift	Scaffold325:3471439
Bta10862	Unknown protein	splice acceptor variant	Scaffold471:791307
Bta11840	Unknown protein	Frame shift	Scaffold52:7764853
Bta12278	Unknown protein	stop gain	Scaffold562:2009445
Bta12668	Unknown protein	start lost	Scaffold607:1307735
Bta12727	Unknown protein	Frame shift	Scaffold607:2833985
Bta13235	Unknown protein	Frame shift	Scaffold64:63239
Bta13327	Unknown protein	splice donor	Scaffold641:3718364
Bta13745	Unknown protein	Frame shift	Scaffold657:1097200
Bta13859	Unknown protein	stop gain	Scaffold67:1393372
Bta13954	Unknown protein	Frame shift	Scaffold699:810303
Bta15302	Unknown protein	Frame shift	Scaffold942:1732675
Bta15415	Unknown protein	Frame shift	Scaffold959:1270849
Bta07758	Zinc finger protein	Frame shift	Scaffold300:1778386
Bta06175	Zinc finger protein 227	stop gain	Scaffold232:1822927
Bta08766	Zinc finger protein 34	Frame shift	Scaffold325:3972542
Bta11305	Zinc finger protein 845	Frame shift	Scaffold493:2884873

### Structural variants

Structural variants were predicted through CNVnator in which the method of detection of structural variants is based on assessing the read of depth of the mapping genome. With CNVnator, among all the structural variants (duplications, deletions, insertions, inversions and translocations), some duplications were detected in the present study. Duplications with more than 1.5 cnv value are enlisted in Table 8 with their positions on the scaffolds and included genes in them. Functional annotations of these genes are presented in Additional file 6. Copy number variations were detected by CNVkit, which are described in Additional file 5. The structural variants in this study is not a comprehensive data and it is necessary to mention that reference genome is a draft genome that is about 90% of total estimated genome (~ 680–690 Mb) and in present study, 88% of this draft genome was covered with mapping reads. When the complete reference genome would be used to detect the structural variants, the results may include some more structural variants.

**Table 8:** Structural Variants

Type	scaffold	start	end	length	CNV	Genes*
duplication	Scaffold112	2,190,001	2,470,000	280,000	1.59861	
duplication	Scaffold130	2,120,001	2,590,000	470,000	1.50412	Bta02314 Bta02317 Bta02318 Bta02321 Bta02311 Bta02319 Bta02313 Bta02315 Bta02320 Bta02322 Bta02312 Bta02316
duplication	Scaffold310	2,080,001	2,870,000	790,000	1.51561	Bta08154 Bta08157 Bta08159 Bta08153 Bta08161 Bta08158 Bta08160 Bta08155 Bta08156
duplication	Scaffold343	3,950,001	4,160,000	210,000	2.19297	Bta09326
duplication	Scaffold403	2,470,001	2,980,000	510,000	1.55897	Bta10316 Bta10315 Bta10317 Bta10318 Bta10319

*Annotation of genes are described in Additional file 6

## Discussion

Whitefly divergence into different distinct genotypes initiates the question whether the divergence results in a complex of different biotypes or it is a complex of different species! In order to resolve the divergence of whitefly question, it would be helpful to set criterion for sorting the different biotypes of whitefly and set a limit above which the difference is sufficient to declare new species status. Biological features e.g. virus transmission capacity, gut microbe diversity, host range, capacity to induce physiological changes in host plants, intermating capabilities, and capacity to spread widely have been used to differentiate cryptic species. Some of the genetic groups share common biological characters and some of the characters also show within group variability. Thus, most of the differences are uninformative or unable to resolve the cryptic species of whitefly. Molecular markers (such as AFLP, RAPD, 16S, CAPS, SCAR and mtCOI) have been used to show genetic differences between genotypes. The 3.5% genetic difference in terms of mtCOI sequences, differentiates almost all reproductively isolated groups according to available biological data. But some reports show disagreements with the outcomes using partial sequences of mitogenome. For example, a recent study [35], using genome wide analysis, suggested that MEAM2 might not be a separate genetic group but fall entirely into the MEAM1 group, whereas previously it was considered as a separate genetic group using mitochondrial genes. A similar phenomenon was observed in a recent study [36], where a combined analysis of experimental biological data with mitogenome sequences proposed that the African silverleafing (ASL) genotype, formerly treated as MED, may form a separate cryptic species. Thus, in view of these reports, species delimitation across the *B. tabaci* species complex requires data in addition to sequence divergence of mtCOI. Many recent reports show that species delimitation of a cryptic species complex requires a multi-method approach that integrates genetic differentiation, biological character, DNA barcoding, molecular phylogenetic analysis and possibly other biological features. In this regard, our study provides whole genome nuclear variants data, which will be useful to improve species delimitation of the *B. tabaci* species complex. It is also necessary to mention that although we detected all these variants between the two species Asia II 1 and MEAM1, but it may also possible that some of the variants may segregate within the same species.

In this study, we have sequenced the genome of the Asia II 1 species of whitefly and have used published transcriptomic data to infer biological differences between Asia II 1 and MEAM1. The sequencing of Asia II 1 not only provided new genomic resources for Asia II 1, but its comparative study with MEAM1 also provided insight into the comprehensive genetic differences between them.

With Blast2GO analysis, high impact variant genes were analyzed to identify the involvement of these genes in molecular pathways. The goal was to find out how genetic variances may alter or affect pathways which may then help in understanding the biological differences between the two species. Signal transduction pathways were considered as one of main points where gene alterations might help the whitefly to deal with any changes in the environment or inside the whitefly cells. Phosphatase and kinases are well-known enzymes in signal transduction pathways [37] as they activate or deactivate the functional proteins by either phosphorylation or dephosphorylation. Kinase and phosphatase functions in antagonistic ways as kinase initiates the phosphorylation and phosphatase removes the phosphate group from its substrate protein. In Additional file 7, it is noticeable that most of the genes are encoding phosphatase and kinase in different pathways e.g. phosphatase in T cell receptor signaling pathways, purine and thymine metabolism, and kinase in drug metabolism (important for pesticide resistance) and phosphatidylinositol signaling pathways. Genetic variants of these genes may alter their systematic regulatory role in biological functions.

Another prominent group of genes comprised “oxidase, dehydrogenase and reductase” enzymes performing functions in oxidative phosphorylation, amino acid (glycine, serine, threonine, valine, isoleucine, arginine and proline) metabolism, steroid degradation and biosynthesis, and biosynthesis of antibiotics. The robustness of a phloem sap sucking pest depends on the amino acid and carbohydrate contents of phloem sap of their host [38] as well as on their processing power of amino acids. For example, a Florida strain of whitefly processes more phloem sap that allows it to have more expanded host range [39]. Phloem sap lacks some essential amino acids and vitamins, so phloem sap sucking pests rely heavily on endosymbionts for some essential amino acids. There are number of genes which are present in more than one pathway for example Bta13274 encodes an oxidase involved in biosynthesis of antibiotics as well as arginine and proline metabolism, indirectly contributes to environmental fitness. A previous study reported that MEAM1 performed better than Asia II 1 on many commonly cultivated crops in China [40], and in another study MEAM1 showed the ability to adapt to unsuitable hosts [41]. Genetic variants in these genes may provide clues to the differential capacity of Asia II 1 and MEAM1 to adapt to changing environments.

Some recent studies report genes showing differential expression upon treatment of insecticide or virus infection. In our data, we identified high impact variants in 14 genes associated with insecticide resistance, 4 genes involved in TYLCV transmission, and 96 genes involved in ToCV transmission. The cathepsin gene family is involved in both insecticide resistance and ToCV transmission. Our results identified high impact variants in cathepsin B (3 genes), cathepsin F (1 gene) and cathepsin L-like genes (3 genes) that are involved in insecticide resistance and ToCV transmission. Cathepsins are proteases involved in many biological functions such as protein degradation, apoptosis, and signaling, and their activity in lysosomes has been broadly connected to virus transmission. The cathepsin B family is expanded in *B. tabaci* and also a novel clade of cathepsin L-like genes is identified in comparison to 15 other arthropods [4] which lead to the prediction of a possible contribution of cathepsin in virus acquisition or other responses that are involved in whitefly-virus interactions. Another important family in which genetic variants were found, associated with insecticide resistance is cytochrome P450 [42]. Two high impact variants were identified in two CYP 450 genes (Bta04696 and Bta06044). Chen et al., [4] inferred the involvement of these genes in insecticide resistance in MEAM1 on the basis of their differential expression upon treatment with insecticides. Another gene that encodes a heat shock protein known to be involved in virus transmission [18] has frame shift variant in Asia II 1. Three genes which have high impact variants and are linked to ToCV transmission are associated with three KEGG pathways: oxidative phosphorylation (Bta05773), T cell receptor signaling pathway (Bta15368), and sucrose and starch metabolism (Bta09856). Bta09856 encodes trehalase a glycosidase which convert trehalose (major sugar reserve in insects play a vital role as an instant source of energy and in dealing with abiotic stresses) into glucose in sucrose and starch metabolism. The inhibition of trehalase causes abnormal growth and unsuccessful stress recovery [43]. Inhibition of trehalase provides promising area towards formulating strategy for insect control. There are also some genes with unknown functions, associated with transmission of ToCV [6]. We reported the genetic variants between Asia II 1 and MEAM1 for these genes, and future annotation of these unknown genes may provide further clues about the mechanism through which whitefly interact with a virus. This comprehensive data set of variations between indigenous and invasive species provide insights into the variations in mechanisms which give different attributes to whitefly species. Based on all these results we conclude that the MEAM1 species is more invasive due to its genetic variations.

## Conclusion

In present study, whole genome wide variants between Asia II 1 (indigenous to the Indian sub-continent and south-east Asia) and MEAM1 (originated in the Middle East but has spread worldwide in recent decades) are presented with their detailed annotations and impact. Variants detection in some important genes such as genes associated with virus transmission and insecticide resistance will help in conceiving future research towards targeted management strategies against whitefly. Furthermore, this study provides a genomic resource of Asia II 1 that will contribute to resolving species delimitation of whitefly.

## Methods

### Colony maintenance and confirmation of cryptic species

The source of whitefly (Asia II-1) population collected from NIBGE, Faisalabad in 2016. An isogenic population was established and maintained in aired glass confinement on cotton (*Gossypium hirsutum*) plants at 32 °C. The universal mtCOI primers C1-J-2195 (5′-TTGATTTTTTGGTCATCCAGAAGT-3′) and TL2-N-3014 (5′-TCCAATGCACTAATCTGCCATATTA-3′ were used to confirm the cryptic species (Asia II 1) [44]. PCR amplifications were performed in 20 μL reactions using DreamTaq Green PCR Master Mix (Thermo Fisher Scientific). The polymerase chain reaction (PCR) cycling parameters were one denaturation cycle of 94 °C for 5 min, followed by 35 cycles of 94 °C for 1 min, 45 °C for 1 min, and 72 °C for 1 min, followed by a final extension of 72 °C for 7 min. PCR products were visualized on a 1% agarose gel. Sanger sequencing [45] confirmed the Asia II 1 culture.

### Genomic DNA extraction and library preparation

DNA extraction was done with “ISOLATE II Genomic DNA Kit” (Bioline Cat No. BIO-52066). Eight libraries with 550 bp insert size were prepared by the Illumina NeoPrep automation system with the library kit, Illumina #NP-101-1001, “TruSeq Nano DNA Library Kit for NeoPrep”, which includes the adapter set “TruSeq LT” (adapter sequences: adapter read1 AGATCGGAAGAGCACACGTCTGAACTCCAGTCA, adapter read2 AGATCGGAAGAGCGTCGTGTAGGGAAAGAGTGT). The target insert size selection was performed by the “Illumina NeoPrep Liberary Prep System”. Actual insert size ranges were calculated by CLC Genomics Workbench (v. 8.5.1).

### Sequencing and mapping with reference genome

Sequencing was performed on the Illumina MiSeq and HiSeq2500 with Rapid v2 chemistry, 2x100bp, across 2 flow cell lanes. The Illumina bcl2fastq v2.16 software was used to convert raw basecalls (.bcl) to fastq.gz, and demultiplex the sequenced pool of libraries by the TruSeq LT indices in the NeoPrep process. The bcl2fastq script was set to automatically trim the adapters, if present. All duplicated reads, low quality regions (phred score less than threshold value) and reads containing N were trimmed. Cleaned reads were mapped onto the total reference genome of whitefly. Reference genome was accessed through ftp://www.whiteflygenomics.org/pub/MEAM1/MEAM1/ [4]. Mapping was done using BWA V0.7.12 with MEM algorithm using CLC Genomics Workbench 7.5. Raw data was visualized and analyzed to pass through quality control steps. Variant calling was performed by Haplotype caller GATK (using ‘ERC GVCF-variant_index_type LINEAR -variant_index_parameter 128,000). Variant filtration was performed two times using parameters (filtration1: DP > 20 & QD > 25.0 & FS < 5.00, filtration 2: DP > 30 & QD > 30.0 & FS = 0.00).

### Analysis of variants

SnpEff [46] was used to annotate variants and effect prediction, and to classify the effects of variants by ‘functional classes’ (missense, nonsense and silent), by ‘impact’ (high, moderate, low and modifier), and by ‘type and region’ (downstream, exon, intergenic, intron, splice site acceptor, splice site donor, splice site region, transcript, upstream, UTR 3′, and UTR 5′). Then all genes that had “high impact variants” were analyzed with “Blast2GO Pro” (trial version) software [47] for gene ontology and to categorize gene functions into three classes: biological process, cellular components and molecular function. With Blast2GO Pro, KEGG pathways of these genes were also developed to analyze their function. All the mapped reads were evaluated to find structural variants. CNVnator [48] was used in the present study to find structural variants. CNVnator analyzes the “read of depth” from alignment to predict the structural variants. Copy number variations were detected by CNVkit [49].

### SNPs validation

Some SNPs were randomly selected for the validation. Eight primer pairs were designed to amplify the regions (each with 500-600 bp length) which have a total of 96 SNPs. DNA was extracted from single whiteflies by the CTAB method [50]. Each region was amplified using DNA extracted from a single whitefly. PCR were performed in 50 μL reactions using DreamTaq Green PCR Master Mix (Thermo Fisher Scientific). PCR cycling parameters were one denaturation cycle of 94 °C for 5 min, followed by 35 cycles of 94 °C for 1 min, 54 °C for 30s, and 72 °C for 40 s, followed by a final extension of 72 °C for 7 min. PCR products were visualized on a 1% agarose gel. Amplified products were purified by “AxyPrep PCR Clean-up Kit” and then these were sequenced by the Sanger method [45]. The sequenced reads were aligned with MEAM1 sequences by DNAStar software to validate the predicted SNPs.

We also analyzed the previously published transcriptomic data of MEAM1 [4, 6, 34]. They reported some genes that were associated with virus transmission (TYLCV and ToCV) and insecticide resistance. In our data we identified genes which had high impact variants and as well as genes previously reported as differentially expressed under virus or insecticide treatment.

## Additional files

Additional file 1: Summary of raw data generated in each library. (XLSX 9 kb)

Additional file 2: Variant statistics. Variants statistics describe number of variants and effects and their types. (XLSX 12 kb)

Additional file 3: BLAST2GO results. Annotations of genes with high impact variants are classified in three main classes (1-biological function, 2-molecular function, 3-cellular components). GO IDs and number of genes in each subclass are also given in this file. (XLSX 22 kb)

Additional file 4: SNP validation. This file describes the positions of amplified regions in scaffolds and position of validated variants in each amplified region. (XLSX 13 kb)

Additional file 5: CNVkit results. (XLSX 19 kb)

Additional file 6: Gene ontology of genes having structural variants. Annotations and InterPro IDs of genes are given. (XLSX 10 kb)

Additional file 7: Gene IDs of genes contributing in different pathways. (XLSX 12 kb)

## Data Availability

The raw data of sequencing reads is submitted to SRA database of NCBI. The data can be accessed with these accession numbers: SRR8656460, SRR8656459, SRR8656466, SRR8656463, SRR8656456, SRR8656455, SRR8656458, SRR8656467, SRR8656465, SRR8656464, SRR8656462, SRR8656461, SRR8656457.

## Abbreviations

BP: Biological Process
CC: Cellular Component
CTAB: Cetyl Trimethyl Ammonium Bromide
GATK: Genome Analysis Tool Kit
INDELS: Insertions Deletions
MEAM1: Middle East Asia Minor 1
MF: Molecular Function
mtCOI: Mitochondrial Cytochrome Oxidase Subunit I
SNP: Singe Nucleotide Polymorphism
ToCV: *Tomato crinivirus*
ToLCHnV: *Tomato leaf curl Hainan virus*
TYLCV: *Tomato yellow leaf curl virus*
